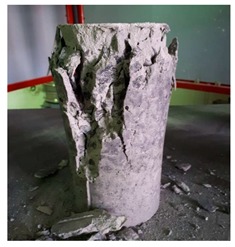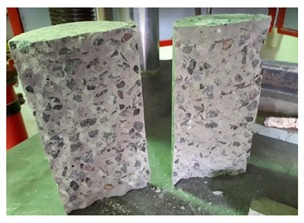# Development of Heavyweight Self-Compacting Concrete and Ambient-Cured Heavyweight Geopolymer Concrete Using Magnetite Aggregates

**DOI:** 10.3390/ma12071035

**Published:** 2019-03-28

**Authors:** Afsaneh Valizadeh, Farhad Aslani, Zohaib Asif, Matt Roso

**Affiliations:** 1 School of Civil, Environmental, and Mining Engineering, University of Western Australia, Perth, WA 6009, Australia; 00100020@uwa.edu.au (A.V.); 22187053@student.uwa.edu.au (Z.A.); 21107373@student.uwa.edu.au (M.R.); 2 School of Engineering, Edith Cowan University, Perth, WA 6027, Australia

**Keywords:** heavyweight self-compacting concrete, geopolymer concrete, heavyweight geopolymer concrete, heavyweight concrete, highly workable, magnetite aggregates

## Abstract

Heavyweight self-compacting concrete (HWSCC) and heavyweight geopolymer concrete (HWGC) are new types of concrete that integrate the advantages of heavyweight concrete (HWC) with self-compacting concrete (SCC) and geopolymer concrete (GC), respectively. The replacement of natural coarse aggregates with magnetite aggregates in control SCC and control GC at volume ratios of 50%, 75%, and 100% was considered in this study to obtain heavyweight concrete classifications, according to British standards, which provide proper protection from sources that emit harmful radiations in medical and nuclear industries and may also be used in many offshore structures. The main aim of this study is to examine the fresh and mechanical properties of both types of mixes. The experimental program investigates the fresh properties of HWSCC and HWGC through the slump flow test. However, J-ring tests were only conducted for HWSCC mixes to ensure the flow requirements in order to achieve self-compacting properties. Moreover, the mechanical properties of both type of mixes were investigated after 7 and 28 days curing at an ambient temperature. The standard 100 × 200 mm cylinders were subjected to compressive and tensile tests. Furthermore, the flexural strength were examined by testing 450 × 100 × 100 mm prisms under four-point loading. The flexural load-displacement relationship for all mixes were also investigated. The results indicated that the maximum compressive strength of 53.54 MPa was achieved by using the control SCC mix after 28 days. However, in HWGC mixes, the maximum compressive strength of 31.31 MPa was achieved by 25% magnetite replacement samples. The overall result shows the strength of HWSCC decreases by increasing magnetite aggregate proportions, while, in HWGC mixes, the compressive strength increased with 50% magnetite replacement followed by a decrease in strength by 75% and 100% magnetite replacements. The maximum densities of 2901 and 2896 kg/m^3^ were obtained by 100% magnetite replacements in HWSCC and HWGC, respectively.

## 1. Introduction

With the advancement in technology and global demand for energy, focus has shifted from traditional energies derived from fossil fuels to non-carbon emitting power generation such as nuclear energy. In addition, the harmful radiation-emitting devices, which threaten human life, had been extensively used with the latest development all over the globe. Heavyweight concrete is broadly utilized material for reactor protecting because of its less expensive and attractive mechanical properties [1,2]. The heavyweight aggregates in concrete plays an imperative role in enhancing solid protecting properties and has been demonstrated to have great shielding properties for lessening photons and neutrons [3,4].

Self-compacting concrete, which is also known as self-consolidating concrete (SCC) is the latest concrete technology that has been used in many high-rise concrete projects all over the globe. SCC is known for its excellent deformability, high resistance to segregation, and successful use in congested reinforced concrete structures characterized by difficult casting conditions that do not allow for vibration [5].

Production of ordinary Portland cement is reported to contribute to 7% of worldwide greenhouse gas emissions, which is equivalent to 1.35 billion tons annually [6]. This is due to the very high temperatures needed to produce it where a majority of the required fuel comes from burning fossil fuels [7]. The calcination of limestone also heavily contributes to greenhouse emissions since it is required in order to produce ordinary Portland concrete (OPC). Future predictions of worldwide concrete demand are set to increase. With the world moving to cleaner energy sources, geopolymer concrete (GC) offers a cleaner and greener way of producing concrete. However, many studies have indicated the potential benefits of fly ash-based GC over the OPC concrete in the last few years [8,9]. Hence, GC has shown the potential to replace OPC by reducing the amount of carbon emissions up to 80% while still maintaining high strength comparable to that of OPC [10]. GC utilizes “end of life” materials such as fly ash and blast furnace slag in its mix design. These materials are currently being disposed of as waste products in industry. Through further utilization, it not only provides economic benefit for producers, but also for those adopting materials in potential mix designs [11]. Since heavyweight concrete is already being used in oil, gas, medical, and nuclear plant applications, a combination of heavyweight and GC could prove to be an environmentally-friendly solution to problems surrounding these industries today. 

## 2. Literature Review

According to the British standards institute [12], the concrete is termed as the heavyweight concrete when the density of concrete reaches an oven dry density of 2600 kg/m³ as opposed to the normal weight concrete, which has a density of 2400 kg/m^3^. The typical aggregates used to develop HWC are magnetite, barite, hematite, limonite, and ilmenite [13]. Magnetite and barite densities are measured to be approximately 30% greater than that of the standard aggregate [14]. Heavyweight classification was achieved with a fine aggregate and course aggregate portions of as low as 850 kg/m^3^ and 1031 kg/m^3^, respectively [15]. Highest densities of 3425 kg/m^3^ were achieved when fine and course aggregate portions were increased to 1400 kg/m^3^ and 1560 kg/m^3^, respectively. However, this will not be achievable in this study due to the flowability requirements of self-compacting concrete [15]. The ordinary sand and fly ash are often suggested as replacements for heavyweight fine aggregate and cement in mixing design of HWC, respectively, since these substitutions are effective in avoiding segregation by adjusting the HWA’s grading and minimizing temperature cracks by lowering heat generated during the hydration process [16].

Ouda [1] investigated the 15 mix designs of high-performance heavyweight concrete using barite, serpentine, magnetite, and goethite for providing radioactive protection, structural integrity, and durability. Ouda [1] examined that high-performance heavyweight concrete containing magnetite aggregates had the maximum compressive strength, which the shielding efficiency increased by raising the fine magnetite aggregate in the mixture, which justifies the use of a magnetite aggregate in this study. Akkurt et al. [17] also examined the attenuation coefficient of HWC using different types of heavyweight aggregates. He revealed that the attenuation coefficient increases with the increase in density of concrete. From multiple studies, it is evidently clear that, as the density and thickness increases, the linear attenuation coefficient of tested samples increases [1,14,15,18,19]. However, due to the brittle failure mode of the concrete samples, premature failure can be a result of cracking formed through voids within the sample. This can be reduced by the inclusion of mineral fillers like fly ash, silica fumes, and ground granulated blast furnace slag that promotes the formulation of a consistent paste while maintaining high compressive strength through the inclusion of chemical admixtures to maintain a constant w/c ratio [20]. Moreover, no correlation has been found to connect a decrease in compressive strength with relation to the proportions of chemical admixtures used [21,22].

SCC was first researched and established in 1988 by Professor Okumara. SCC is highly flow-able and high resistance to segregation concrete that can fill in formwork under its own weight without using vibrators or other mechanical means [23]. The method of SCC is different from OPC with respect to two unique properties, flowability, and high segregation resistance [24]. To enhance the flowability of SCC, a low yield stress and moderate viscosity are required to ensure non-segregation in aggregates during flow [25]. However, segregation resistance in SCC is developed due to high viscosity [26]. For a concrete mix to be classified as self-compacting, the parameters for slump flow and J-ring tests need to be fulfilled [27,28]. Ingredients such as fly ash and ground granulated blast furnace slag (GGBFS) can be used as a replacement in order to increase flowability [28]. Excessive amounts of cement in the mix may lead to excessive slump loss and, by substituting fly ash and GGBS for cement, it makes the concrete more economical to implement in the industry [23,27]. Aslani et al. [29,30] found a significant improvement in rheological behavior of concrete mixes by using chemical admixtures. The superplasticizer admixture helps increase flowability of the mix. However, excessive use will lead to bleeding [21]. Viscosity modifying agents (VMA) are then used to reduce bleeding and to form a consistent and homogenous mix. On the other hand, a high water reducer agent (HWRA) significantly improves the cement dispersion and provides flowable concrete with greatly reduced water demand [22].

Su et al. [27] examined the increase in compressive strength of SCC by increasing cement and silica fume content in concrete. However, medium strength SCC can be obtained with cementitious values as low as 200 kg/m^3^ [20]. Bouzoubaâ and Lachemi [28] investigated the compressive strength and flowability of concrete with varying fly ash proportions. They found that incorporating higher levels of fly ash could maintain compressive strength while increasing flowability of the mix. 

GC, which is also known as alkali-activated or inorganic concrete, is a different kind of concrete, which uses different chemistry to that found in OPC concrete. The historical backdrop of the geopolymer started with the first patented by a German chemist and engineer Kűhl in 1908, where a combination of alumina and silica content (vitreous slag) with alkali (alkali sulphate or carbonate) source lead to develop solid material comparable to OPC [31]. However, in 1979, Joseph Davidovits gave the name to aluminosilicate-based material as the term geopolymer and he defined the geopolymer as short-range order inorganic polymer that forms when high concentrated aqueous alkali hydroxide-silicate solution is added to the alumino-silicate materials [32]. The main difference from regular concretes containing Portland cements and GC is that the GC does not form calcium silicate hydrates for strength but instead make use of poly-condensation of silica, alumina, and high alkali contents to achieve adequate structural strength [7]. The properties of the geopolymer relies on many factors such as the chemical composition of the binders, the type of alkali activators, the concentration of the activator, curing condition, and water content [33].

Currently, the most common binder used in the formation of the geopolymer is fly ash. The fly ash geopolymer has proved to have better mechanical properties and durability when compared to OPC [7,34]. Fly ash-based geopolymer mixes performed significantly better at higher temperatures [33,35,36]. Moreover, the longer setting time and lower strength under ambient conditions make plain fly ash-based geopolymers impracticable for field use [37]. Notable studies have been done on the fly ash geopolymer combined with some additional materials [38,39]. The inclusion of GGBFS into fly ash-based geopolymers resulted in quicker setting times and higher strength, which results in creating a concrete suitable for ambient curing conditions [37,40]. Nath et al. [41] investigated the properties of fly ash-based GC with the different proportions of additives such as GGBFS, OPC, and calcium hydroxide (CH). The maximum compressive strength was achieved with the 10% addition of GGBFS in fly ash-based geopolymer after 28 days at ambient curing conditions.

Alkaline solution plays an important role in the geopolymerization process [42]. Different alkali activators have been used in GC such as alkali hydroxides, alkali silicates, alkali carbonates, and alkali sulphate [31]. Currently, alkali silicate is an important chemical compound that has been used as a good activator to a binder and plays an important role in the performance of GC. Na-silicates are most often used activators because of their low cost compared to K-silicate solutions. Many researchers have developed GC by combining an activator between alkali silicates and alkali hydroxides with appropriate ratios [43]. Palomo et al. [44] found a compressive strength of ranges between 35 to 40 MPa, which will be produced from a reaction of different fly ash with an alkali activator (NaOH) of ranges from 8 to 12 mol/L cured at 85 °C in 24 hours, while the compressive strength (with the same conditions) will increase up to 90 MPa when alkali silicate (Na_2_SiO_3_) was added with the ratio of SiO_2_/Na_2_O = 1.23. Similarly, Aslani [45] found significant compressive strength by using alkaline solution prepared with a ratio of Na_2_SiO_3_/NaOH = 2.5, which further justifies the use of sodium hydroxide with sodium silicate as the alkaline solution. 

The strength development of the GC is heavily influenced by the water content [37]. Shayan and Pahedonous [43] investigated the compressive strength with the increase of water content in the geopolymer. They found that the compressive strength decreased exponentially when the water to solid ratio increased from around 0.15 to 0.5. Hence, increasing the water content led us to increase the slump flow, which, in turn, decreased the mechanical properties i.e. compressive strength. Admixtures can be used to improve and manipulate the fresh properties of a concrete mix to better suit the concrete for a specific use [46,47]. The addition of superplasticizers can see the early strength of concrete increase by 50% to 75% and an increase in workability on the fresh properties of concrete [48]. Although the mechanical properties and fresh properties of GC are heavily influenced by the types of binders and activators used, the ratio of the binder to the activator can also significantly impact the characteristics of a mix [37]. An activator-to-binder ratio should lie between 0.3–0.5 [41]. Hamidi et al. [49] observed an increase in compressive strength of 10 MPa when the activator-to-binder ratio increased from 0.25 to 0.3.

## 3. Research Significance

The goal of this study is to create two preliminary mix designs with the first being a concrete that both possesses heavyweight and self-compacting attributes through the addition of magnetite as the heavyweight aggregate at various percentages of total aggregate replacement. The second being an ambient-cured heavyweight geopolymer concrete (HWGC) incorporating magnetite at various percentages of replacement. This research will aim to examine the fresh and mechanical properties of both heavyweight self-compacting concrete (HWSCC) and HWGC mixes. For heavyweight self-compacting concrete, research will be focused on achieving the greatest concrete density while maintaining flow requirements in order to achieve self-compacting classification. Investigations involving GC will aim to produce a concrete of adequate density while maximizing compressive strength. The hardened property investigation includes compressive, tensile, and flexural strength testing of both types of concrete in this research. 

## 4. Experimental Study

### 4.1. Materials

#### 4.1.1. Binders for Heavyweight Self-Compacting Concrete

##### Cement

Cement used in this study consisted of general-purpose cement (GPC) that conforms to the Australian Standard [50]. It conforms to all Australian requirements that allow it to be used with fly ash, blast furnace slag, and chemical admixtures [51,52,53]. The properties of GPC can be seen in Table 1.

##### Fly Ash

Fly ash used as the partial replacement of cement in this study was selected due to its ability to promote flow-ability and increases in compressive strength due to their spherical glassy shape particles. Fly ash conforms to requirements of [53] and properties of fly ash can be found in Table 1.

##### Ground Granulated Blast Furnace Slag

Ground Granulated Blast Furnace Slag (GGBFS) was selected as the partial replacement of cement due to its ability to increase compressive strength. GGBFS used in this study as another supplementary cementitious material and conform to Australian Standard [52]. The properties are shown in Table 1.

##### Silica Fume

Silica fume was selected in this mix design due to its ability to increase compressive strength while minimizing fresh property performance of the cement paste and conform to the Australian Standard [54]. The silica particles are finer than the cement particles. However, silica fume helps in getting high early strength gain, low concrete permeability, and reduce the probability of bleeding in concrete mix. The properties of the silica fume are shown in Table 1.

#### 4.1.2. Binders for Heavyweight Geopolymer Concrete 

##### Fly Ash

The primary binder material used for GC was fly ash, which provides strength and improves the workability of concrete due to their spherical glassy shape particles. Fly ash used for GC produced in this study are the same as that used for HWSCC. The properties can be seen in Table 1.

##### Ground Granulated Blast Furnace Slag

The addition of GGBFS into fly ash-based GC increases the strength and reduces the setting time, which results in developing a concrete suitable for ambient curing [37]. GGBFS complies with [52]. The properties are shown in Table 1.

##### Alkaline Solution

The alkaline solution was prepared by adding sodium hydroxide (NaOH) and sodium silicate $\left(Na_{2}SiO_{3}\right)$ solutions. Sodium hydroxide liquid was prepared in the laboratory by mixing 99% pure sodium hydroxide pellets, purchased from local producer, with normal tap water. The N-grade sodium silicate solution used in this study, collected from a local producer, had a molecular ratio of SiO_2_ to Na_2_O of 3.2 with a 1.39 g/cc density (SiO_2_ = 28.6%, Na_2_O = 8.9%, and H_2_O = 62.5% by weight). The purpose of the sodium silicate solution in GC is combined with sodium hydroxide to form a geopolymer paste that acts like an alkaline activator and binds all aggregates and unreacted materials [6].

#### 4.1.3. Aggregates for HWSCC and HWGC

##### Natural Normal-Weight Aggregates

**Figure 1:**
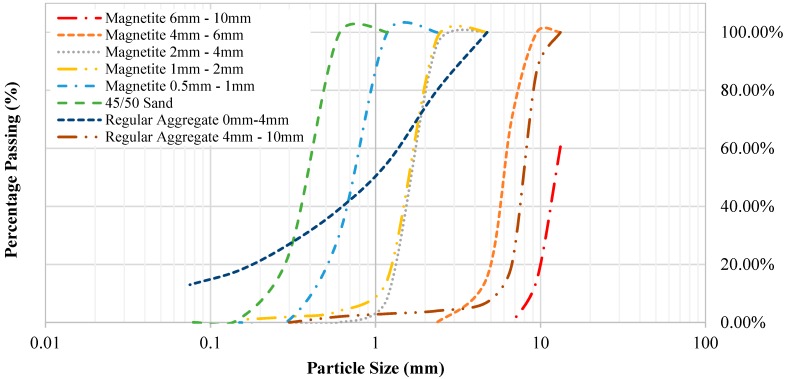
Grading curve of aggregates and sand.

In this study, regular aggregates (normal weight) were used in control mixes for HWSCC and HWGC and typically consist of crushed stone. The natural crushed 0–4 mm and 4–10 mm aggregates were used as natural fine and coarse aggregates, respectively. Fine AFS 45–50 silica sand obtained from Rocla Quarry Products, Western Australia was used in this study. The sampling methods and testing of these aggregates were done according to AS 1141 [55]. The properties of sand and natural crushed aggregates are shown in Table 2. The output of the particle distribution curve is shown in Table 3 and the graphical curve is shown in Figure 1.

##### Heavyweight Magnetite Aggregates

**Figure 2:**
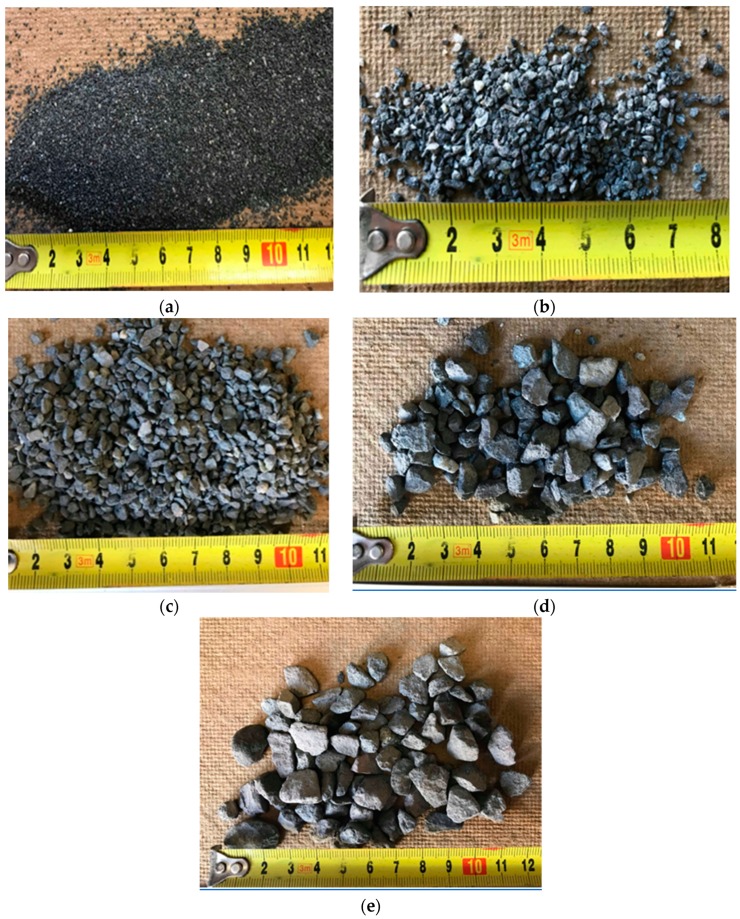
Magnetite aggregates (**a**) 0.5–1 mm, (**b**) 1–2 mm, (**c**) 2–4 mm, (**d**) 4–6 mm, and (**e**) 6–10 mm.

Heavyweight aggregate that was used in both HWSCC and HWGC mixes consisted of magnetite. Magnetite is a low grade and unrefined iron ore with a density of approximately 1.4 times that of regular aggregates. Magnetite was chosen due to its availability in Western Australia as well as its ability to maintain high compressive and tensile strength. The properties of magnetite aggregates are shown in Table 4. Sizes were broken down into the following categories, 0–1 mm, 2–4 mm, 4–6 mm, and 6–10 mm. Particle sizes from 0–6 mm were fine aggregates and sizes from 6–10 mm were considered coarse aggregates. Particle size distribution can be seen in Figure 1 and Figure 2 and Table 3.

#### 4.1.4. Chemical Admixtures

Chemical admixtures were used in both HWSCC and HWGC mixes. The main advantages of chemical admixtures are that the fresh properties on the mix can be modified without having to alter the w/c ratio or quantity of dry ingredients. The superplasticizer admixture (SP) was used, which satisfies Type SN chemical admixture, according to AS-1478.1 [56]. It is designed to improve the flow properties of concrete by lowering the viscosity and yield stress of fresh concrete. The high-range water reducer agent (HRWRA) and the viscosity modifying agent (VMA), which were used in this study, according to AS-1478.1 [56]. The significant improvement of the rheological behavior of GC was observed with the addition of these admixtures in this experimental study. However, SP increases workability of the mixture without compromising strength and HRWR increases the flowability of the mix by mimicking higher water contents without adding additional water. Lastly, VMA improves viscosity of the mix, which should start to segregate [26].

### 4.2. Mix Designs

#### 4.2.1. Self-Compacting Control Mix Design and Heavyweight Self-Compacting Concrete 

In this study, the HWC mixes were developed based on the American Concrete Institute method of absolute volume stablished for normal concrete and has been proven as the more convenient approach for producing heavyweight concrete [57,58]. A standard self-compacting control mix (CM1) was prepared by using natural fine and coarse aggregates only, and three HWSCC mixtures HWSCC1, HWSCC2, and HWSCC3 were prepared by replacing natural crushed aggregates with heavyweight magnetite aggregates at 50%, 75%, and 100% by volume, respectively. In total, four mixes were developed to investigate the performance of standard SCC and HWSCC mixes. The mix design set was based on the control mix, which contains a water-to-binder ratio of 0.45 and a total cementitious material content of 585 kg/m³ with appropriate chemical admixtures to fulfil adequate requirements for SCC. The binder composition of the mix was composed of 51.5% cement, 25.7% fly ash, 17.2% GGBFS, and 5.6% silica fume. However, binder/aggregate ratio of 0.3 was obtained. Mix design for control HWSCC can be seen in Table 5. Once a HWSCC control mix (CM1) was developed, then three heavyweight mixes designs were formulated including each containing incrementally more magnetite replacement in order to investigate the effect of aggregate replacement of fresh and hard properties. HWSCC1 contained 50% magnetite aggregate replacement. HWSCC2 had 75% magnetite replacement while HWSCC3 contained 100% magnetite aggregate replacement. Binder content was kept the same as the control mix design and admixtures were altered accordingly to attain self-compacting applications. Mix designs for HWSCC can be seen in Table 6.

#### 4.2.2. Geopolymer Control Mix Design and Heavyweight Geopolymer Concrete 

The control mix design of GC is based on the foundations of a previous study conducted by Nath et al. [41]. The standard geopolymer control mix was developed by using natural coarse aggregate only while three HWGC mixes were prepared by replacing natural coarse aggregate with heavyweight coarse magnetite aggregate in a control mix design. In this study, the standard geopolymer control mix (CM2) includes 400 kg/m^3^ binder content with natural crushed aggregates and a water-to-binder ratio of 0.123, which is less than that suggested to achieve minimum needed workability. However, the sodium hydroxide solution is made by combining pure solid sodium hydroxide pellets with water and this gives water content 0.2 times the total binder within the whole mix to make up the minimum needed to achieve workability and maintain adequate compressive, tensile, and flexural strengths. The binder composition of the mixes was composed of 90% fly ash and 10% GGBFS. Alkaline solution was used as 40% of the total binder and the ratio of sodium silicate to sodium hydroxide was kept constant at 2.5. The concentration of sodium hydroxide was 14 moles in all mixtures. The CM2 mix design can be seen in Table 7.

The HWGC mixes (HWGC1, HWGC2, and HWGC3) were developed by substituting coarse aggregates with magnetite aggregates at 50%, 75%, and 100% by volume, respectively, and can be found in Table 8. However, these three mixtures were conducted with the same binder content, alkaline solution, and chemical admixtures such as in a standard geopolymer control mix.

### 4.3. Preparation of Ingredients 

#### 4.3.1. Heavyweight Self-Compacting Concrete

Since ingredients for heavyweight self-compacting concrete mixes were found to not be porous in nature, there was no need to pre-soak or provide any additional preparation to aggregates or binders.

#### 4.3.2. Heavyweight Geopolymer Concrete

Prior to mixing for HWGC mixes, the alkaline solution was prepared by mixing sodium hydroxide and sodium silicate solutions alone and left in room temperature to cool down for 1 h. First, the sodium hydroxide solution of 14 mole concentration is prepared by mixing pure sodium hydroxide pellets in water. Then, sodium silicate solution is mixed in sodium hydroxide solution and was allowed to cool down for 1 h since it releases heat because of the exothermic reaction [59]. Since aggregates were the same as that in HWSCC mixes, no prior preparation was needed for the magnetite aggregate or regular aggregate.

### 4.4. Mixing and Casting Method

During the mixing processes of HWSCC and HWGC mixes, it is very important to persist in being a uniform technique of production for each mix to minimize the chance of errors in terms of reactions between the materials [26]. In order to achieve consistency throughout all mixes, the mixing procedure was done, according to the parameters outlined in AS1012.2 [60].

In HWSCC mixes, first, all the aggregates and cementitious materials were put into the mixer pan in sequence from a large size material to a smaller size material and then dry mixed for 5 min. The next step is to mix them for another 3 min after adding a specified amount of water. Then HRWRA and SP admixtures were added in and mixed for another minute. Lastly, the VMA admixture was added as needed to achieve SCC requirements. Another 2 min of mixing was done before testing fresh properties and molding the specimens.

In HWGC mixes, all saturated surface dry aggregates, sand, and solid binder materials were collected in the pan mixer and then dry mixed for up to 5 min. Once the solid materials were mixed comprehensively, then the alkaline solution was added and followed by water and superplasticizer and allowed to mix for another 5 min.

The specimens’ cast used in this study consisted of 100 × 200 mm cylinders to determine the compressive and tensile strengths. On the other hand, rectangular prism molds of 450 × 100 × 100 mm in dimensions were casted to determine the flexural strength. Molds were lightly brushed in oil before concrete was poured inside. In order to achieve homogeneity and eliminate voids, concrete was added in small portions and allowed to settle. Molds were removed 24 hours following pouring and samples were then placed in a control room at a temperature of 20 ± 2 °C for 7 and 28 days until it was time for mechanical tests.

### 4.5. Test Procedure

#### 4.5.1. Fresh Property Testing

##### Heavyweight Self-Compacting Concrete

The fresh properties of HWSCC mixes are assessed through the tests described under the guidelines and SCC criteria defined by EFNARC [61] and AS-1012.3.5 [62]. Fresh property tests conducted for HWSCC mixes involved slump flow tests incorporating T500 measurements and J-Ring tests. These tests determine the viscosity, flowability, passing ability, and resistance to segregation. Slump flow, T500, and J-ring tests were conducted using the Abram cone in accordance with AS-1012.3.5 [62]. The slump flow test was recorded by measuring the diameter flow diameter and the time to reach 500 mm (T_500_) was also measured. J-ring tests were recorded by measuring the diameters and J-ring height differences.

##### Heavyweight Geopolymer Concrete

Fresh properties testing for HWGC mixes were limited to slump flow testing and it was not designed to be self-compacting. The slump flow test was conducted using the Abram cone in accordance with AS 1012.3.5 [62]. The slump flow diameter was recoded for all mixes.

#### 4.5.2. Mechanical Properties Testing

In this study, the hardened mechanical properties of both HWSCC and HWGC were examined by compressive strength, tensile strength, flexural strength, and stress-strain behavior. Three representative concrete specimens from each batch were tested at 7 and 28 days.

The compression testing procedure follows AS1012.14 [63]. The three 100 × 200 mm cylindrical specimens were loaded at a rate of 0.2 kN/s until failure. However, the splitting tensile strength was conducted in accordance with AS1012.10 [64] on three 100 × 200 mm cylindrical specimens for testing ages of 7 and 28 days. The specimens were loaded at a rate of 1.5 ± 0.5 kN/min until failure [26]. Moreover, three cylindrical samples were weighed and their dimensions were measured to obtain their hardened density in accordance with AS1012.12.1 [65]. These testing processes were followed for all mixes.

The flexural strength test was conducted in accordance with AS1012.11 [66]. Three prisms of size 450 × 100 × 100 mm were tested under four-point loading to measure the flexural strength. The maximum load at failure was used to calculate the flexural strength of the sample. This testing process was used for all mix samples. Moreover, one of the prisms from each batch for flexural testing was attached with 60 mm horizontal and 60 mm vertical strain gauges to examine the flexural displacement of all concrete mixes in accordance with AS1012.17 [67].

## 5. Results and Discussion

### 5.1. Fresh Properties

#### 5.1.1. Heavyweight Self-Compacting Concrete

Fresh property testing is at the core of any SCC mix. It needs sufficient flowability so that the concrete can uniformly distribute around the designated area under its own weight without any additional compaction or vibration [26,29]. The incorporation of heavyweight aggregate with a higher density than that of regular aggregate requires adjustments in the quantity of admixtures to ensure SCC’s flowability. All HWSCC mixes tested fulfilled self-compacting requirements outlined in EFNARC [61]. However, both HWSCC and HWGC mixes also satisfy the requirements of heavyweight density in accordance with BS EN-206 [12] where densities should be greater than 2600 kg/m^3^.

Experimentation has shown it is possible to develop HWSCC mixes containing magnetite aggregates satisfying requirements outlined in EFNARC [61]. The slump flow and J-ring diameters are reduced with the increase in magnetite replacement since flowability of the mix decreased due to a large difference in specific gravity of magnetite aggregates. Hence, this decrease can also be attributed to the difference in the water absorption rate in normal-weight coarse aggregates and in magnetite aggregates, where the latter absorbs more water than normal-weight coarse aggregates. The large difference in specific gravities of aggregates in concrete is a primary cause of segregation within concrete [68]. The chemical admixtures were adjusted in order to maintain SCC properties as well as avoiding segregation within concrete. Therefore, the amount of SP and VMA admixtures were increased with the increase in magnetite aggregates in concrete mixes.

**Figure 3:**
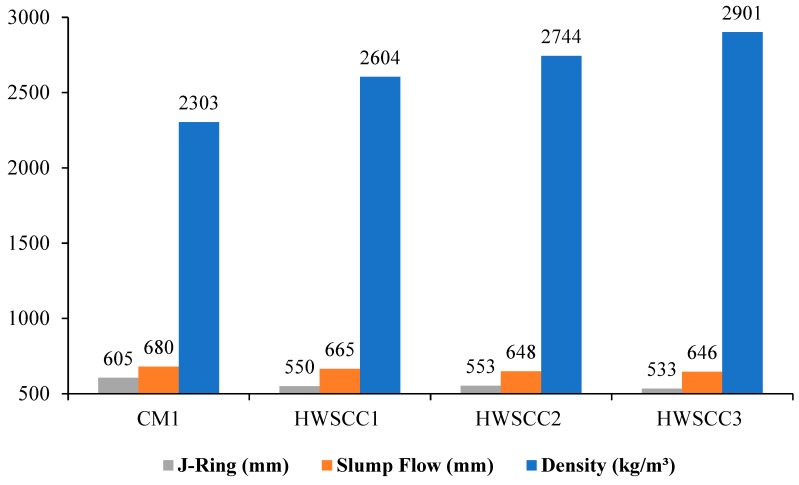
Fresh property results for HWSCC mixes.

The outcomes of densities and fresh properties of CM1 and HWSCC mixes tested by the slump flow test (diameter and T500 time) and J-Ring test (diameter, center height, ring inside and outside depths) are mentioned in Table 9 and Figure 3. In J-ring tests, the center height and the depth inside and outside the ring were increased with the increase in magnetite replacements. This can be attributed to the large difference in the specific gravities and water absorption rates of natural aggregates and magnetite aggregates. Moreover, the inclusion of different proportions of chemical admixtures in HWSCC mixes may have affected the J-ring measurements. Although the depths and center heights of HWSCC1, HWSCC2, and HWSCC3 were almost the same but, overall, the increase in depths and heights were exhibited by HWGCC mixes when compared to CM1.

#### 5.1.2. Heavyweight Geopolymer Concrete

In this experimental study, HWGC mixes were not designed to be self-compacting. Therefore, only a slump flow test was conducted to examine the workability of concrete. According to ASTM C143/M-03 [69] and Ghosh and Ghosh [70] in its table of GC workability criterion, the slump flow for a high workability GC ranges >250 mm. However, the CM2 and HWGC mixes satisfied the geopolymer criterion for flowability. Moreover, these mixes showed a decrease in the slump flow diameter with an increase in magnetite aggregates, as seen in HWSCC mixes. This decrease can be attributed to the difference in the specific gravities and water absorption of aggregate replaced in this study, where the magnetite aggregates have a higher rate of water absorption than natural crushed aggregates, which results in a decrease in slump flow.

**Figure 4:**
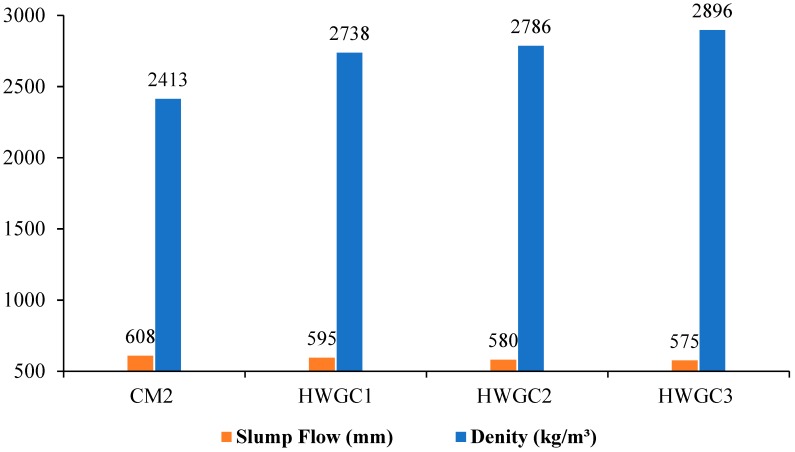
Fresh property results for HWGC mixes.

The results of fresh property testing of CM2 and HWGC mixes, including slump flow diameter with respective dry densities following the hardening of samples, are presented in Table 10 and Figure 4.

### 5.2. Mechanical Properties 

#### 5.2.1. Compressive Strength

**Figure 5:**
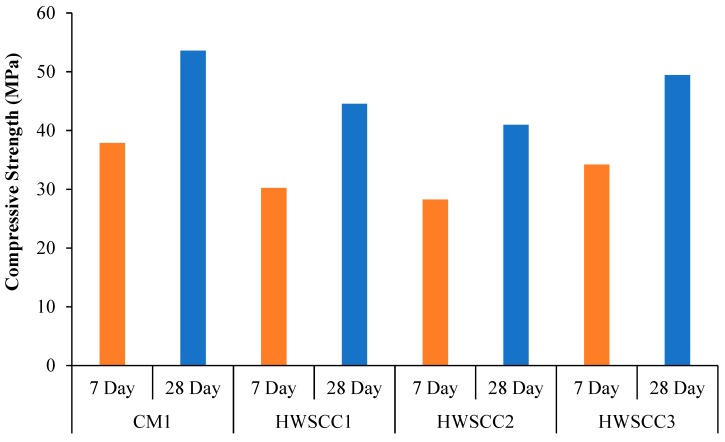
Compressive strength of HWSCC mixes.

**Figure 6:**
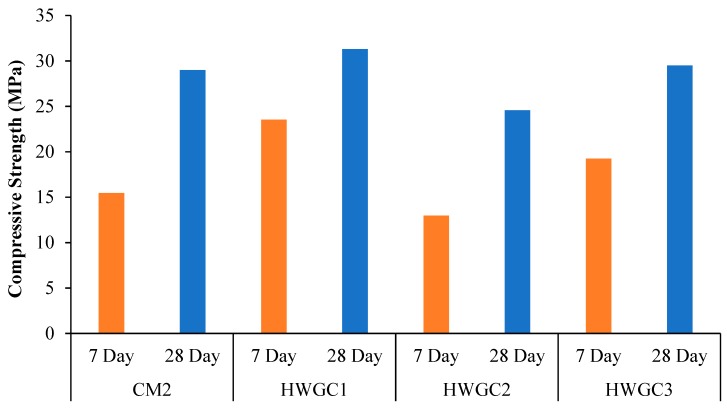
Compressive strength of HWGC mixes.

Compressive strength of HWSCC and HWGC mixes containing 50%, 75%, and 100% magnetite replacements in their control mixes are displayed in Figure 5 and Figure 6, respectively.

From Figure 5, experimentation investigation into the compressive strength of HWSCC incorporation magnetite aggregates has yielded results indicating that the magnetite replacement into self-compacting control mix acts to decrease the compressive strength of concrete. However, it was found that the compressive strength increases with curing time for all hardened mixes. This is attributed to the increased content of hydration products leading to an increase of compressive strength. Overall, the highest compressive strength was achieved in CM1 with 0% aggregate replacement. On the other hand, the lowest compressive strength was achieved by HWSCC2 with 75% aggregate replacement after 28 days of ambient temperature curing.

Since these results indicate a drop in compressive strength from CM1 containing regular aggregate to the HWSCC mixes containing magnetite aggregate, it is possible that the significant difference of densities and improper interlocking of the regular and magnetite aggregates lead to a reduction of compressive strength. In addition, the heavyweight aggregate ratios have shown an inverse proportion to the compressive strength due to the interaction between cement paste and the heavyweight aggregate, which causes weak adhesion between paste and structure of heavyweight aggregates [71]. The previous study has shown that the compressive strength decreased with an increase of the heavyweight aggregate [72,73]. Thus, the compressive strength recorded from HWSCC1, HWSCC2, and HWSCC3 mixes containing 50%, 75%, and 100% magnetite aggregates resulted in the compressive strength of 16.9%, 23.5%, and 7.7% lower than that of the maximum strength obtained from CM1 after 28 days, respectively. However, the 75% replacement of magnetite aggregate in the control mix has revealed the substandard distribution of aggregates, which results in a suboptimum mix. This led to the lowest compressive strength among other HWSCC mixes. Therefore, a conclusion can be drawn that the inclusion of heavyweight aggregate to a CM1 mix decreases compressive strength, which is consistent with researched literature, but 75% replacement of magnetite aggregate is not optimal in mix design, which has shown the lowest strength among all HWSCC mixes. Compressive strength results for HWSCC can be seen in Figure 5. 

In HWGC mixes, the compressive strength was considerably lower than that of HWSCC mixes and it can be found in Figure 6. Although the strength was lower, different environmentally-friendly binding materials were used in the GC as compared to SCC mixes. Similar to a compressive strength trend in HWSCC mixes, the HWGC2 mix containing 75% replacement revealed the lowest compressive strength as compared to other HWGC mixes containing 50% and 100% magnetite replacements. This has proven that the 75% magnetite aggregate with 25% natural aggregate in concrete mix is an inferior aggregate proportion for compressive strength.

In HWGC mixes, a different trend was observed similarly to HWSCC mixes since the reactions of the fillers and activators in GC might have affected the behavior of HWGC with the aggregate replacements. With the 50% magnetite replacement, the compressive strength was increased as compared to the CM2 mix’s compressive strength of 29 MPa, which is followed by a decrease in compressive strength with 75% magnetite replacement. The HWGC2 mix containing 75% magnetite aggregate revealed the lowest compressive strength of 24.56 MPa. However, with the 100% magnetite replacement, the HWGC3 mix showed almost the same strength as in the CM2 mix. Compressive strength results for all HWGC mixes can be seen in Figure 6.

#### 5.2.2. Tensile Strength

**Figure 7:**
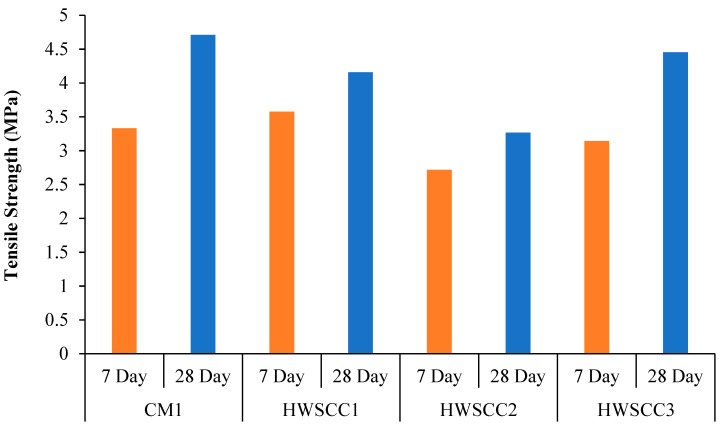
Tensile strength of HWSCC mixes.

Results of experimental investigation of the magnetite replacement on HWSCC mixes are shown in Figure 7. Magnetite replacement into the SCC mix on average show a decrease in tensile strength. However, the same trend was examined by increasing magnetite replacement such as in compression strength results of HWSCC mixes. This decrease in tensile strength can be attributed to the significant difference of densities and improper interlocking of the regular and magnetite aggregates, which leads to a reduction in tensile strength. The results revealed that, in all mixes of HWSCC, the maximum tensile strength was achieved in a control mix containing 0% magnetite aggregates after 28 days. The strength of all mixes is significantly reduced by magnetite replacements of 50% and 75%, which is followed by an intense increase at HWSCC3 containing 100% magnetite replacement. The tensile strength of 4.45 MPa was achieved by HWSCC3 mix at 28-day testing. However, the reduction in tensile strength of 11.7%, 30.6%, and 5.5% were examined as compared to control CM1 mix strength of 4.71 MPa with 50%, 75%, and 100% magnetite replacements, respectively. The lowest tensile strength of 3.27 MPa was recoded in HWSCC2 with 75% replacement of heavyweight aggregate, which is consistent with compressive strength results.

**Figure 8:**
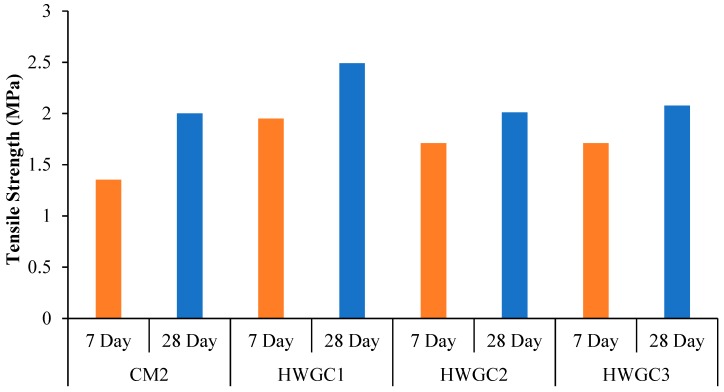
Tensile strength of HWGC mixes.

With respect to an increase in magnetite replacement in HWGC mixes, the trend of tensile strength was similar to that of the HWGC compressive strength result and it can be seen in Figure 8. An increase in tensile strength was noted when magnetite aggregate increased from 0% to 50%. This was followed by a decrease in tensile splitting strength when magnetite replacement increased to 75% and 100%. This decrease in strength can be attributed to the poor interaction between cement paste and a heavyweight aggregate, which causes weak adhesion between paste and structure of heavyweight aggregates. However, the tensile splitting strength of HWGC1 is 24.5% higher than the CM2 mix under ambient temperature curing after a 28-day age, which is consistent with HWGC compression results. However, the tensile strength is 19.3% and 16.5% lower than the HWGC1 strength with the increase of magnetite aggregates by 75% and 100%, respectively. The lowest tensile splitting strength of 2.01 MPa was observed with 75% magnetite replacement. 

#### 5.2.3. Flexural Strength

**Figure 9:**
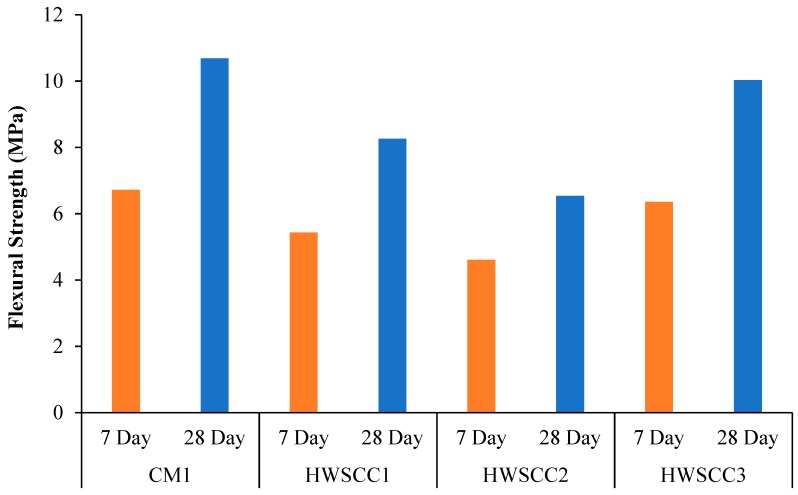
Flexural strength of HWSCC mixes.

**Figure 10:**
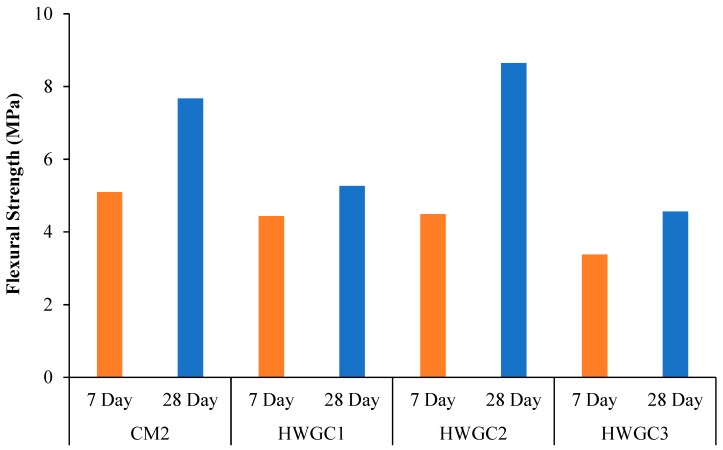
Flexural strength of HWGC mixes.

Figure 9 and Figure 10 show the effect of magnetite replacement on flexural strength of HWSCC and HWGC mixes after 7 and 28 days, respectively. Trend found in HWSCC mixes seem to follow that of compressive and tensile results, where the highest strength were exhibited in control mixes for HWSCC containing only regular aggregate. The maximum flexural strength of 10.68 MPa was achieved by the CM1 mix. However, the flexural strength recorded for CM1 was only 6% higher than that of HWSCC3 containing 100% heavyweight aggregate replacement, which failed at about 6.72 MPa. Moreover, the lowest flexural strength for HWSCC were observed in HWSCC2 containing 75% replacement of heavyweight aggregate, which is consistent with compressive and tensile strength results and only reaches about 4.61 MPa. This can be attributed to the substandard distribution of aggregates at 75% magnetite replacement, which results in a suboptimum mix. This led to the lowest flexural strength among other HWSCC mixes. The flexural strength of 5.43 MPa was examined by HWSCC1 containing 50% magnetite aggregates.

In HWGC mixes, a different trend for flexural strength was observed in compressive and tensile results in HWGC and can be attributed to the change in loading direction while applying flexural load, which shows different behavior of HWGC mixes, as examined in the compression and tension. However, HWGC2 mixes containing 75% magnetite replacement achieved the maximum flexural strength of 8.65 MPa. Moreover, the lowest flexural strength recorded for GC was 4.56 MPa in HWGC3 containing 100% heavyweight aggregate replacement. The flexural strength of HWGC mixes can be found in Figure 10.

**Figure 11:**
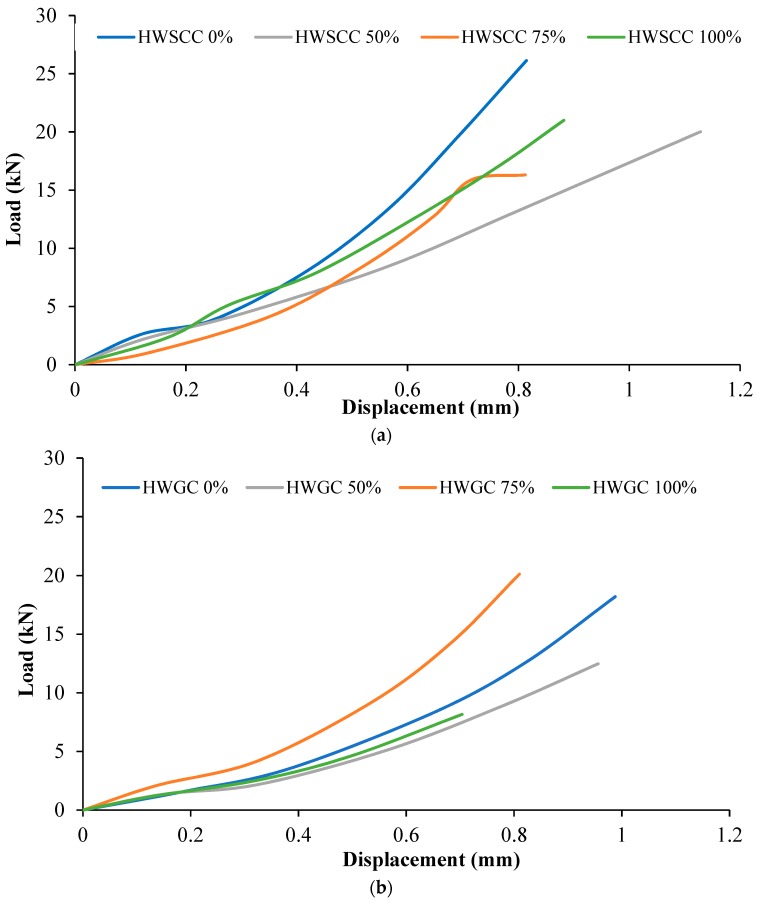
Flexural load-displacement curves of (**a**) HWSCC and (**b**) HWGC mixes.

Flexural displacements were recorded for both HWSCC and HWGC mixes and results can be seen in Figure 11. As can be seen in Figure 11a, HWSCC with 50% heavyweight aggregate replacement recorded the highest displacement of 1.11 mm with a respective load of almost 20 kN. However, HWSCC with 0% aggregate replacement recorded the highest flexural strength but with the lowest corresponding flexural displacement. HWSCC with 75% magnetite aggregate recorded the lowest flexural load of around 16 kN only with corresponding flexural displacement of 0.8 mm.

In HWGC mix, the control GC mix containing 0% magnetite replacement showed the maximum flexural displacement of almost 1 mm. HWGC 100% replacement mix revealed the lowest flexural load of around 8 kN with the lowest flexural displacement of 0.7 mm. The results of flexural displacement for HWGC mixes can be seen in Figure 11b.

#### 5.2.4. Failure Modes

Table 11 shows the failure modes for compressive and tensile tests in all concrete mixes. A vast majority of compressive testing resulted in compressive failure of the sample, which leaves a cone-shaped structure that could be removed from the sample. This conical failure mode is most preferred and was expected from previous literature [26,29]. HWSCC almost consistently showed conical failure patterns. The figures of all HWSCC showed conical failure mode and it can be seen in Table 11. However, HWGC mixes tended to show less uniformity of cracking from compressive testing. In HWGC mixes, conical failure and shear failure modes were observed by compression testing. Different mode of failures, other than the conical failure pattern, would suggest premature failure of the sample. Thus, it shows low strength in concrete. The shear failure of HWGC2 mix can be seen in Table 11.

Tensile failure modes were consistent throughout both heavyweight self-compacting and heavyweight geopolymer concrete mixes, which leaves a crack through the center of the sample where the loading bar was applied.

## 6. Conclusions 

This experimental study hoped to develop and contribute to research into the use of heavyweight aggregates in self-compacting and geopolymer concrete. The following conclusions can be drawn based on information acquired through this study.

- As the percentage of heavyweight aggregate is increased into the HWSCC and HWGC mixes, the density of the mix increases due to the magnetite aggregate having a higher density. Densities increased from 2300 kg/m^3^ with 0% HW aggregate replacement to 2900 kg/m^3^ for 100% aggregate replacement.
- In HWSCC and HWGC mixes, as the percentage of magnetite aggregate increases, the flowability and pass ability of the mix decreases. This can be seen in fresh property testing where slump and J-Ring diameters decrease, as well as an increase in the center height in J-Ring results. This decrease can also be attributed to the large difference in the specific gravities and water absorption rate in normal-weight coarse aggregates and that in magnetite aggregates, where the latter absorbs more water than normal-weight coarse aggregates. However, the slump flow diameters decreased by 2.2%, 4.7%, and 5% with magnetite replacement of 50%, 75%, and 100% in the control SCC mix. On the other hand, the slump flow diameters decreased by 7.75%, 5.3%, and 6.1% with magnetite replacement of 50%, 75%, and 100% in a control GC mix.
- Since these results indicate a drop in compressive, tensile, and flexural strengths from control SCC mix containing regular aggregate to the HWSCC mixes containing magnetite aggregate, it is possible that the significant difference of densities and improper interlocking of the regular and magnetite aggregates lead to a reduction in compressive strength. In addition, the heavyweight aggregate ratios have shown inverse proportion to the compressive and tensile strengths due to the poor interaction between cement paste and heavyweight aggregate, which causes weak adhesion between paste and structure of heavyweight aggregates.
- Similar to compressive and tensile strength trends in HWSCC mixes, the HWGC mix containing 75% replacement revealed the lowest compressive and tensile strength as compared to other HWGC mixes containing 50% and 100% magnetite replacements. This has proven that the 75% magnetite aggregate with 25% natural aggregate in concrete mix is an inferior aggregate proportion for compressive strength.

## Figures and Tables

**Table 1:** Properties of cement, fly ash, ground granulated blast furnace slag, and silica fume.

General Purpose Cement	Value	Fly Ash	Value
**Chemical Properties**		**Chemical Properties**	
CaO	63.40%	CaO	3.30%
SiO_2_	20.10%	SiO_2_	50.40%
Al_2_O_3_	4.60%	Al_2_O_3_	31.50%
Fe_2_O_3_	2.80%	Fe_2_O_3_	10.40%
SO_3_	2.70%	SO_3_	0.10%
MgO	1.30%	MgO	1.10%
Na_2_O	0.60%	Na_2_O	0.30%
Total Chloride	0.02%	K_2_O	0.50%
**Physical Properties**		SrO	<0.1%
Specific Gravity	3.0–3.2 t/m^3^	TiO_2_	1.90%
Fineness index	390 m^2^/kg	P_2_O_5_	0.50%
Normal consistency	27%	Mn_2_O_3_	0.20%
Setting time initial	120 min	Total Alkali	0.60%
Setting time final	210 min	**Physical Properties**	
Soundness	2 mm	Relative Density	2.29
loss on ignition	3.80%	Moisture	<0.1%
Residue 45 μm sieve	4.70%	Loss on Ignition	1.10%
**Mechanical Properties**		Sulphuric Anhydride	0.10%
Mortar Comp Str.		Chloride Ion	0.00%
*f’_c_* 3 Days	38.6 MPa	Chemical Composition	92.30%
*f’_c_* 7 Days	48.4 MPa	Relative Water Requirement	93%
*f’_c_* 28 Days	58.5 MPa	Strength index	102%
Shrinkage 28 days	640 μ strain		
**Ground Granulated Blast Furnace Slag**		**Silica Fume**	
**Chemical Properties**		**Chemical Properties**	
S	0.40%	Silicon as SiO_2_	98%
SO_3_	2.40%	Sodium as Na_2_O	0.33%
MgO	5.70%	Potassium as K_2_O	0.17%
Al_2_O_3_	12.60%	Available Alkali	0.40%
FeO	0.80%	Chloride as Cl^−^	0.15%
MnO	0.10%	Sulphuric Anhydride	0.83%
Cl	0.01%	Sulphate as SO_3_	0.90%
Insoluble residue content	0.20%	**Physical Properties**	
**Physical Properties**		Bulk Density	625 kg/m^3^
Specific Gravity	3.0–3.2	Relative Density	2.21
Relative Water requirement	103%	Pozzolanic Activity at seven days	111%
Relative Strength	100%	Control Mix Strength	31.3 MPa
Temperature rise	18.8 °C	Moisture Content	1.10%
Fineness (passing 45 μm)	98%	Loss of Ignition	2.40%

**Table 2:** Properties of sand and natural crushed aggregates.

AFS 45-50 Silica Sand	Value	Natural Crushed Aggregates	Value
**Chemical Properties**		**0–4 mm**	
SiO_2_	99.86%	Apparent Particle Density	2.76 t/m^3^
Fe_2_O_3_	0.01%	Particle Density Dry	2.65 t/m^3^
Al_2_O_3_	0.02%	Particle Density SSD	2.69 t/m^3^
Cao	0.00%	Water Absorption	1.40%
MgO	0.00%	Moisture Content	2.50%
Na_2_O	0.00%		
K_2_O	0.00%	**4–10 mm **	
TiO_2_	0.03%	Moisture Content	0.5%
MnO	<0.001%	Flakiness Index	24.0%
**Physical Properties**			
Loss on Ignition	0.01%		
Water Content (@105^o^C)	<0.001%		
AFS Number	47.50%		

**Table 3:** Particle distribution of all aggregates.

Characteristic Sieve Size (mm)	Percentage Passing (%)							
	AFS 45/50 Silica Sand	Heavyweight Aggregates (Magnetite)					Natural Aggregates	
		(0.5–1) mm	(1–2) mm	(2–4) mm	(4–6) mm	(6–10) mm	(0–4) mm	(4–10) mm
13.2	-	-	-	-	100.0	60.0	-	100.0
9.5	-	-	-	-	100.0	14.7	-	87.0
6.7	-	-	-	-	70.3	0.0	-	20.0
4.75	-	-	100.0	100.0	15.7	-	100.0	7.0
2.36	-	100.0	98.0	95.8	0.0	-	80.0	4.0
1.18	100.0	98.0	14.7	8.5	-	-	55.0	3.0
0.6	99.0	14.7	3.9	0.0	-	-	39.0	2.0
0.3	23.8	3.9	2.0	-	-	-	27.0	0.0
0.15	1.3	2.0	1.0	-	-	-	18.0	-
0.75	0.0	-	0.0	-	-	-	13.0	-

**Table 4:** Properties of magnetite aggregate.

Properties	Specific	Value
Chemical	Fe	>95.5%
	Si	2.20%
	C	0.50%
	Mn	2.20%
Physical	Hardness	5.1
	Specific Gravity	4.6 g/cm^3^

**Table 5:** HWSCC control mix design.

Mix	w/b	Cement	Fly Ash	GGBFS	Silica Fume	Fine Aggregate (0–4) mm	Sand	Coarse Aggregate (6–10) mm	Binder/Agg
**CM1**	0.45	300	150	100	33	1050	360	900	0.3

All quantities are in kg/m^3^, SP = 2.5 l/m^3^, HRWRA = 0.75 l/m^3^, VMA = 0.

**Table 6:** HWSCC mix designs.

Ingredient	HWSCC1	HWSCC2	HWSCC3
GP Cement	300	300	300
Fly Ash	150	150	150
GGBFS	100	100	33
Silica Fume	33	33	33
Water	262.35	262.35	262.35
0–1 mm HWA	173.5	259.88	346.5
1–2 mm HWA	173.5	259.88	346.5
2–4 mm HWA	173.5	259.88	346.5
0–4 mm RA	525	262.5	0
4–6 mm HWA	225	337.5	450
6–10 mm HWA	225	337.5	450
4–100 RA	450	225	0
45/50 Sand	360	360	360
SP (L/m^3^)	2.75	3.25	3.75
HRWR (L/m^3^)	0.75	0.75	1.125
VMA (L/m^3^)	0	0.375	2.5

HWA = Heavyweight Aggregate, RA = Regular Aggregate, Quantities in kg/m^3^ unless noted otherwise.

**Table 7:** HWGC control mix design.

Mix	w/c	Fly Ash	GGBFS	Sodium Silicate	Sodium Hydroxide	Sand	Course Aggregate	Water
**CM2**	0.123	360	40	114.3	45.7	650	1210	49.23

All quantities are in kg/m^3^, SP = 3.28 l/m^3^, HRWR = 0, VMA = 0, 16.4 kg solid sodium hydroxide and 29.21 l water for 14 mole concentration.

**Table 8:** HWGC mix designs.

Ingredient	HWGC1	HWGC2	HWGC3
Fly Ash	360	360	350
GGBFS	40	40	40
Sodium Silicate Solution	114.3	114.3	114.3
Sodium Hydroxide Solution	45.7	45.7	45.7
Water	49.23	49.23	49.23
45/50 Sand	650	650	650
6–10 mm HWA	860	1289	1692
4–10 mm RA	614	307	0
SP (L/m^3^)	3.28	3.28	3.28
HRWR	-	-	-
VMA	-	-	-

HWA = Heavyweight Aggregate, RA = Regular Aggregate, Quantities in kg/m^3^ unless noted otherwise, 16.4 kg solid sodium hydroxide and 29.21 l water for 14-mole concentration.

**Table 9:** Fresh property results of HWSCC mixes.

Fresh Property	CM1	HWSCC1	HWSCC2	HWSCC3
Density (kg/m^3^)	2303	2604	2744	2901
Slump Diameter (mm)	680	665	648	646
T_500_	<1	<1	<1	<1
J-Ring Diameter (mm)	605	550	553	533
Center Height (mm)	34	40	45	41
Depth Inside J (mm)	29	37	38	37
Depth Outside J (mm)	21	26	25	26

**Table 10:** Fresh property results of HWGC mixes.

Fresh Property	CM2	HWGC1	HWGC2	HWGC3
**Slump Flow (mm)**	612.5	565	580	575
**Density (kg/m^3^)**	2412.63	2738.01	2785.89	2896.36

**Table 11:** Failure modes.

Mix	Compressive	Tensile
CM1	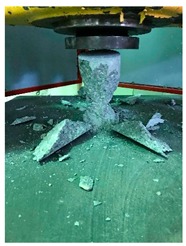	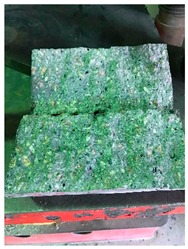
HWSCC1	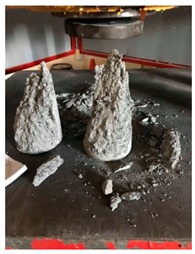	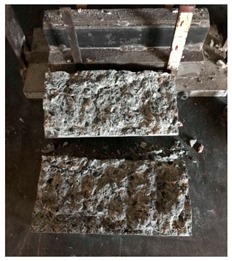
HWSCC2	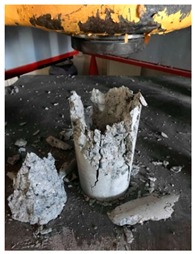	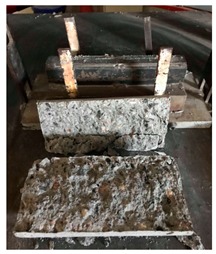
HWSCC3	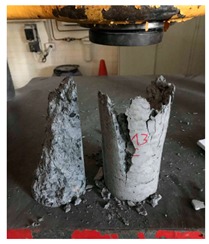	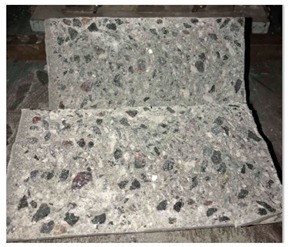
CM2	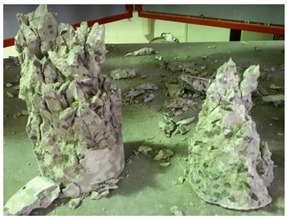	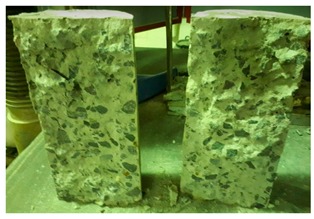
HWGC1	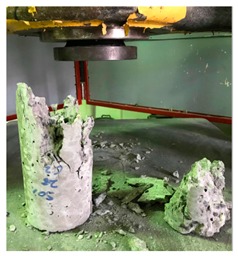	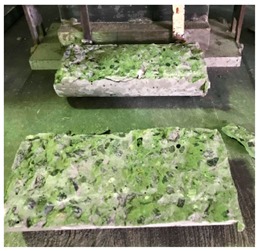
HWGC2	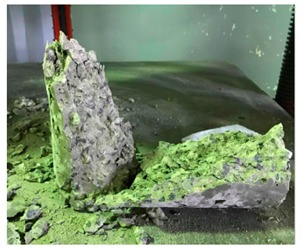	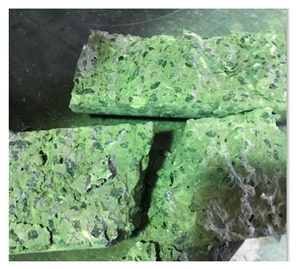
HWGC3